# Rheological and Mechanical Characterization of Asphalt Binder Modified with Plastic Waste Polymers

**DOI:** 10.3390/polym18131574

**Published:** 2026-06-24

**Authors:** Yerzhan Imanbayev, Yerdos Ongarbayev, Ainur Zhambolova, Yernar Kanzharkan, Aliya Kenzhegaliyeva, Zhannur Myltykbayeva, Uzilkhan Yensegenova, Akkenzhe Bussurmanova, Anar Akkenzheyeva

**Affiliations:** 1 Institute of Combustion Problems, Almaty 050012, Kazakhstan; erdos.ongarbaev@kaznu.edu.kz (Y.O.); zhambolova.ainur@mail.ru (A.Z.); erxank@mail.ru (Y.K.); aliakenzhik@gmail.com (A.K.); 2 Faculty of Chemistry and Chemical Technology, Al-Farabi Kazakh National University, Almaty 050040, Kazakhstan; zhannur.myltykbaeva@gmail.com (Z.M.);; 3 Engineering Faculty, Yessenov University, Aktau 130003, Kazakhstan; akkenzhe.bussurmanova@yu.edu.kz (A.B.);

**Keywords:** bitumen, modification, polymer waste, rheology, microscopic study, asphalt concrete

## Abstract

Asphalt concrete pavements in many regions suffer from premature deterioration caused by low-temperature cracking and rutting resistance under heavy traffic loads and high summer temperatures. While polymer-modified bitumen is widely used to improve pavement performance, the high cost of commercial polymers restricts its extensive application. This study evaluates the potential of polymer waste as an alternative modifier for asphalt binders to enhance mechanical performance while reducing economic and environmental costs. Experimental results demonstrate that an optimal plastic waste content of 1.0–1.5% significantly improves rutting resistance and increases binder rigidity. The incorporation of 1.5% low-density polyethylene (LDPE) and high-density polyethylene (HDPE) enhances deformation resistance, elastic modulus, and temperature stability. LDPE exhibits better compatibility with bitumen and dissolves more readily, contributing to improved binder homogeneity, whereas HDPE provides higher stiffness and thermal stability. The combined use of polymer waste with styrene–butadiene–styrene (SBS) produces a pronounced synergistic effect, leading to improvements in physical and mechanical properties exceeding 25% compared to Kazakhstan regulatory standards. Increasing polymer waste content further enhances the rigidity of both the binder and asphalt concrete, thereby improving rutting resistance and plastic deformation at elevated temperatures. The proposed approach offers a cost-effective and sustainable solution for road construction, promoting plastic waste recycling, reducing reliance on virgin polymers, and improving pavement durability, particularly under the climatic and traffic conditions of Kazakhstan.

## 1. Introduction

Kazakhstan is located in three road-climate zones and has a predominantly sharply continental climate. High summer temperatures require measures against the formation of waves and lagoons on the surface, and low winter temperatures cause thermal cracking. Frequent temperature transitions through 0 °C also complicate the work of asphalt concrete. An effective way to improve the performance of bitumen and asphalt is to modify them with polymer additives that increase heat resistance, lower brittleness temperature, and extend the material’s operating range [1,2,3].

The modern road should allow cars to reach their design speed at maximum safety. Traffic safety and design speed are the most generalized and significant performance indicators of a road surface, which is largely determined by the properties of asphalt concrete, namely its resistance to the formation of tracks, roughness and brightness. Ensuring a high level of performance for asphalt and concrete pavement not only improves road safety but also makes traffic more comfortable, which is particularly important nowadays. The performance properties of asphalt concrete are primarily related to the quality of the type of mineral material used in asphalt concrete. Asphaltic concrete is a multi-component conglomerate system in which the mineral shell as individual grains is in an environment that bonds these grains into a single monolith. In this regard, the structure of asphalt concrete is characterized not only by the size, shape, quantitative ratio of grains of different sizes, and properties of mineral components but also by the structure and properties of bitumen and the nature of interaction between it and the grains of the mineral composition [4,5,6,7,8].

The main disadvantage of asphalt concrete as a road construction material is its high temperature dependence on its strength and deformation properties. The increase in temperature causes a decrease in the viscosity of the bitumen contained in asphalt concrete, which leads to reduced strength and an increase in deformation. Rapid industrial growth has made plastic waste pollution a serious problem for both human health and the environment. In most cases, the lack of a policy for sorting and recycling them in landfills in many countries results in highly contaminated mixed plastic waste. Kazakhstan is one example. To ensure the required quality of plastic materials for certain purposes, manufacturers use different types of polymers and additives [9,10,11,12]. This makes it possible to invent a more efficient alternative lightweight material to existing ones in order to reduce carbon footprints and energy consumption. On the other hand, it complicates waste composition [13]. Although recycling is the best option among all existing methods today in terms of environmental sustainability, efforts to find new energy solutions lead to the production of poorly recycled materials, which makes it impossible to create a single processing system. When plastic waste from different sections is mixed due to the difficulty of separating and identifying the type of plastic after use, and it is contaminated with dirt, it typically has poor recyclability [14]. In this case, carefully separated mixed plastic waste is crucial; otherwise, many polymers give low properties compared to the original product [6,15].

Despite the many advantages that modern technologies in the production of modified binders provide, these materials can increase the total cost of asphalt by up to 40%. Road engineers are actively trying to use waste as a modifier of bitumen in order to improve productivity and reduce the cost of road materials. Thus, when laying 1 km of road up to 1 t of polymer waste can be disposed of by modifying the bitumen material. To solve the problem of polymer waste, various studies have been carried out on its recycling and secondary use in the construction of asphalt concrete coatings. Most research focuses on the use of polymer wastes as modifiers of bitumen to improve the fatigue strength, rigidity, fluidity and strength of asphalt mixtures [16,17,18,19]. For polymer–bitumen binders, the cost directly depends on the amount of polymer added, while the amount of polymer added affects the quality of the finished polymer. Currently, most of the world’s consumption of bitumen still comes from basic bitumen. Even for one country, the percentage fluctuates in different years. According to data published by the European Asphalt Association, the percentage of use of polymer–bitumen in all road construction used annually has generally been less than 20% in most European countries over the last 3 years [20].

The paper [21] presents the influence of the type and content of plastic on the mechanical and various properties and durability of a hot asphalt concrete mixture. The optimal plastic content is 3%, 6%, 9% and 12% of the binder. A stability and fluidity test in Marshall showed that stability and fluidity values improved with increased plastic content. The 9% LDPE mixture showed a maximum stability of 12 kN for the Marshall test.

The research [22] investigated the effect of the use of hybrid elastomer and polymers on the rheological properties and chemical relationship of an asphalt binder. The results of the study show that hybrid polymers have a significant impact on improved properties of modified asphalt, including fatigue cracking and the formation of rutting resistance.

A morphological analysis of LDPE and LLDPE modified binders showed low phase separation (5%), and HDPE-modified binders (13%) and PP-modified binders showed high phase separation (20%). Polymer additives have demonstrated a significant improvement in the phase dispersion of binders [23].

In laboratories, bituminous polymer binders are usually obtained under “ideal” conditions, namely when mixing for a long time is carried out, using highly effective dispersing agents, with precise temperature control, etc. Transferring this technology to the current production of an asphalt concrete plant is not always possible. When modifying bitumen, it is necessary to adjust the technology to the properties of a specific base bitumen; at the plant, performance is also critical and often time-limited due to concerns about bitumen oxidation. There are no methods of rapid control of the morphology of modified bitumen. As a result, there is often a decrease in the homogeneity of polymer–bitumen binders, and to achieve the required binding quality, the polymer content has to be increased, which increases the cost of the final product. Our research goal includes studying the impact of household polymer waste on the modification of bitumen and asphalt concrete mixtures utilized as ecofriendly plastic wastes, reducing the cost of the modified bitumen binders. The obtained results show a positive effect of polymer waste on the quality of polymer–bitumen binders.

## 2. Materials and Methods

The Aktau bitumen plant provided road bitumen 70/100 for the study of the modification of bitumen with polymer waste. Samples of polymer waste were taken from the recycling workshops. Table 1 shows the physical and mechanical characteristics of secondary polyethylene products of different densities. The polymer waste was produced by mechanical recycling. Table 1 shows that secondary polyethylene loses its basic properties but retains fairly high strength and deformation performance.

The experimental test plan included sample preparation, modification conditions, testing procedures, and evaluation criteria (rheological characterization, morphological analysis, and asphalt concrete performance testing).

**Figure 1:**
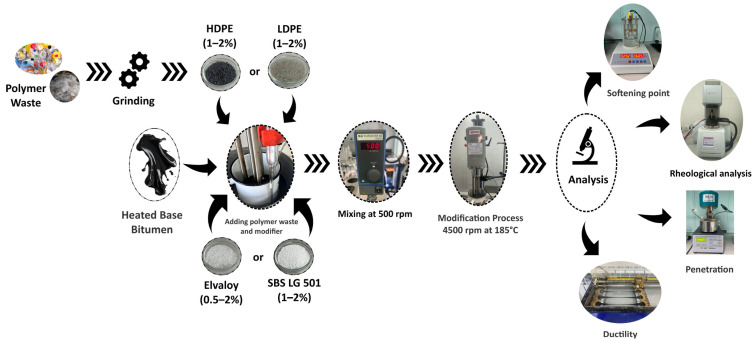
Modification of bitumen with polymer waste.

The modification of bitumen with polymer waste was carried out as follows (Figure 1): The base bitumen was heated in the reactor at 110 °C, and then a certain amount of polymer waste (from 1 to 2%) and ready-made polymer modifiers were added and then mixed into a homogeneous state of the mixture at 500 rpm. Then the temperature was raised to 185 °C, and the sample was mixed in a shear mixer at a speed of 4500 rpm for 3 h. Process temperature selection is justified for the dissolution of finished polymers and reaction modification of SBS polymers.

The main properties of the modifiers SBS LG 501 (LG Chem, Seoul, Republic of Korea) and Elvaloy (Dow Inc., Houston, TX, USA) are presented in Table 2. The density of SBS is 0.94 g/cm^3^, and the melt flow index shows no more than 0.5 g/10 min. The butadiene-to-styrene ratio is 69/31%. This parameter is used to assess the rubber’s homogeneity, as the content of bound styrene affects its physical and mechanical properties.

The Elvaloy modifier provides high rutting resistance. It has been shown that depending on the dosage, Elvaloy increases the plasticity range of bitumen binders by up to 30 °C. Road bitumen modified with Elvaloy polymers provide superior adhesion to mineral materials compared to unmodified bitumen. The melt flow index is 8 g/10 min, which indicates how easily a thermoplastic material can flow under a given pressure and temperature.

Penetration characterizes the degree of bitumen hardness and is determined by a penetrometer. Penetration is determined by the penetration depth in the bitumen of a standard needle with an area of 0.1 mm^2^ under a load of 100 g for a time of 5 s at 25 °C. Penetration values were determined using a penetrometer, in accordance with Kazakhstan standard 1226 [24].

Ductility also indirectly characterizes the adhesion of bitumen. It is generally accepted that the stretchier bitumen, the higher the cracking of asphalt concrete, especially at low temperatures. Ductility values were determined by using a ductilometer apparatus, in accordance with Kazakhstan standard 1374 [25].

The softening temperature of a bitumen binder is used to determine the resistance of the bitumen to deformation and, together with the depth of penetration, characterizes its thermal sensitivity. Road bitumen is a thermoplastic material. The softening point was determined using the ring-and-ball method, in accordance with Kazakhstan standard 1227 [26].

Penetration, ductility, and softening point are the main parameters used to evaluate the properties of bitumen and polymer–bitumen binders. Penetration characterizes the bitumen binder’s rigidity, and a lower value of the binder shows that it is more rigid and resistant to deformation. The ductility value reflects the bitumen binder’s ability to stretch without breaking and is related to crack resistance at low temperatures. The softening point determines the bitumen binder’s heat resistance to prevent rutting resistance and its ability to maintain shape at high temperatures. These parameters allow us to evaluate the balance between the bitumen material’s rigidity, elasticity, and heat resistance. Modified bitumen with polymers leads to a decrease in penetration value, an increase in the softening point, and a change in ductility, which typically depend on the type of polymer. This combined analysis presents the improved performance properties of polymer–bitumen binders and their suitability for use under variable temperatures and heavy traffic loads.

SEM images of the modified bitumen were filmed under a Leica DM 6000M optical microscope (Leica, Stockholm, Sweden) at Farabi University.

A number of road construction specialists consider that the standard requirements for binders are not fully in line with modern requirements [27]. Therefore, along with standard methods, the rheological properties of bitumen were investigated using the SmartPave 102e (Anton Paar, Graz, Austria) rheometer, using an 8 mm diameter parallel plate geometry at 25 °C.

Temperature sweeps were conducted on a dynamic shear rheometer (DSR) at a constant angular frequency of 10 rad/s. Tests were performed over a temperature range of 4 to 76 °C with a 6 °C increment. For temperatures of 28 °C and above, 25 mm-diameter plates with a 1 mm gap were used. At each temperature increment, the key rheological parameters of the bitumen binder were recorded, including the complex shear modulus (G*) and phase-shift angle (δ). The obtained data were used to evaluate the temperature sensitivity, elasticity, and viscous properties of the bitumen binder samples.

To construct master curves for the rheological properties of bitumen, binders were prepared at temperatures of 30, 40, 50, 60, and 70 °C using a parallel plate system. Before measurements, the samples were placed between the rheometer plates and trimmed to remove excess material from the plate edges and ensure accurate measurement of the gap. A frequency sweep was performed in the angular frequency range from 100 to 0.1 rad/s on a logarithmic scale. Tests were conducted at a constant strain amplitude of 0.1%, ensuring that the material operated in the linear viscoelastic region. Each temperature step included 18 measurement points. Master curves were constructed using temperature–frequency superposition with the data normalized to a reference temperature of 40 °C. The Arrhenius model implemented in the SmartPave 102e software was used to calculate the temperature shift coefficients. Based on the obtained coefficients, the horizontal shear coefficient (a_T_) was plotted as a function of temperature. The shear coefficient values were used to combine the experimental curves obtained at different temperatures into a single master curve.

**Figure 2:**
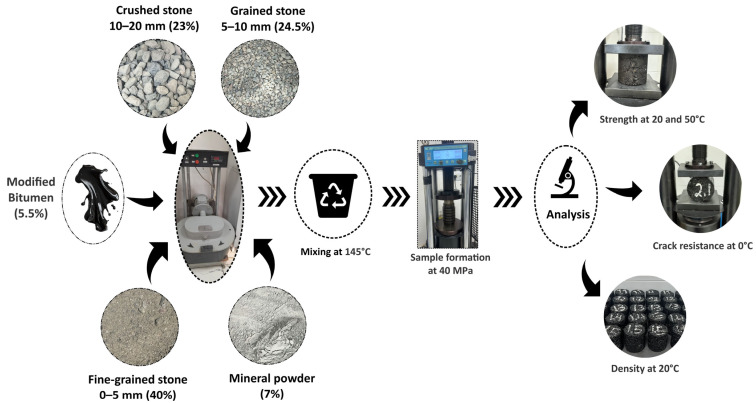
Preparation of laboratory asphalt concrete samples.

Two types of asphalt concrete (in accordance with Kazakhstan standard 1218-2024 [28]) were prepared for testing (Figure 2): one containing modified bitumen and the other neat bitumen (control sample). The materials were mixed using the hot wet-mixing method in two stages. In the first stage, crushed stone and fine-grained stone (5 mm) were heated in an MLA-20 laboratory (Novoe delo, Saint Petersburg, Russia) mixer at 160–170 °C, and then the crushed stone, fine-grained crushed stone, and mineral powder were dry-mixed for 1 min. Then, in the second stage (wet mixing), while mixing the mineral materials (crushed stone, fine-grained crushed stone and mineral powder), the bitumen binder was preheated to 145 °C and added and mixed for 3 min. The homogeneity degree was assessed visually based on the degree of grain coverage with the bitumen binder. The temperature of the asphalt concrete was 150–160 °C. Then asphalt concrete samples were transferred to a cylindrical form that was 71.4 mm in height and diameter and compacted under a constant load of 40 MPa. After 3 min, the load was removed, the sample was removed from the form using a compactor, and its height was measured with a caliper, with an accuracy of no more than 0.1 mm. The samples were kept in an air-dry condition for 24 h.

## 3. Results and Discussion

In Kazakhstan road bitumen is traditionally produced using direct oxidation of heavy petroleum residues. This method is characterized by insufficient resistance to oxidative aging processes and poor adhesion to acidic minerals, leading to leaching of individual road pavement particles under loads as early as the first year of operation. An effective way to improve the properties of bitumen is to incorporate polymers. However, local asphalt concrete producers clearly lack experience and knowledge in working with polymer modifiers that vary in nature and properties. Table 3 presents the physical and mechanical properties of the polymer–bitumen binders prepared with LDPE polymer waste and the SBS and Elvaloy modifiers. Without the SBS modifier, the penetration value is reduced, the ductility is lower, and the softening temperature is slightly increased. Adding the 1% SBS modifier leads to penetration-value increases, but the softening temperature and ductility remain approximately the same. A further increase in SBS content to 2% increases the softening temperature, while penetration increases and then decreases. This is explained by the fact that the presence of plastic waste in the system leads to the complete distribution of the SBS polymer in the bitumen and increases strength. A polymer–bitumen binder with 1.5% polymer waste and 1.5% SBS modifier meets all parameters of Kazakhstan standard 2534-2014 [29] for the bitumen modified polymer (BMP) 50/70.

By increasing the amount of polymer waste, a decrease in the softening point is observed, which characterizes the material hardness. These data support the hypothesis of a gradual increase in bitumen plasticity range in the presence of the Elvaloy modifier, which is associated with the formation of new structures within the product. The penetration value and ductility decrease but then increase. This is explained by the fact that the introduction of polymer waste into the bitumen increases the viscosity of the system, which reduces the penetration value. Polymer waste can act as a filler and reduce the surface force of the bitumen binder, which reduces the ductility of the product. Polymer–bitumen binders with 1, 1.5, and 2% LDPE waste and 0.5–1% of the Elvaloy modifier meet the requirements of Kazakhstan standard 2534-2014 for BMP 35/50.

Table 4 presents the HDPE waste penetration value and ductility of the bitumen binder, which decrease as the softening temperature increases. The addition of the modifier at an amount of 1% leads to a decrease in the penetration and ductility values, but the softening temperature remains approximately the same. A further increase in the modifier to 1.5% leads to an increase in the softening temperature, while the penetration and ductility value increase but then decrease. The polymer–bitumen binder with 1% HDPE waste and 1.5% of the SBS modifier satisfies the requirements of Kazakhstan standard 2534-2014 for BMP 35/50.

According to the experimental data the optimal receipt formulation for preparing polymer–bitumen binders using plastic waste involves up to 2% waste and 0.5 to 1.5% polymer modifier. The advantage of using polymer waste is its low cost of production. The structure formation of a bitumen binder with the SBS and Elvaloy modifiers leads to a reduction in the delamination of polymer waste in the bitumen composite.

It should be noted that despite the widespread use of polymer–bitumen binders in road construction, the issues of bitumen modification and the influence of the morphology of modified bitumen on the technological properties of asphalt concrete remain unclear. A large number of specialists are currently involved in studying the properties of polymer–bitumen binders. However, the primary focus is on the rheology of polymer-modified bitumen, with less attention paid to morphology [19].

At room temperature and under actual operating conditions, polymer-modified bitumens typically represent micro- or macro-heterogeneous systems as composite materials. Their properties are determined by the phase structure of the mixture, particularly mechanical properties primarily, and the properties of the continuous phase. Therefore, only those polymers that form the continuous phase within the composition possess the ability to impart elasticity to bitumen. The role of the polymer, forming the dispersed phase within the bitumen mass, is limited to strengthening the material by filling it with particles [17].

**Figure 3:**
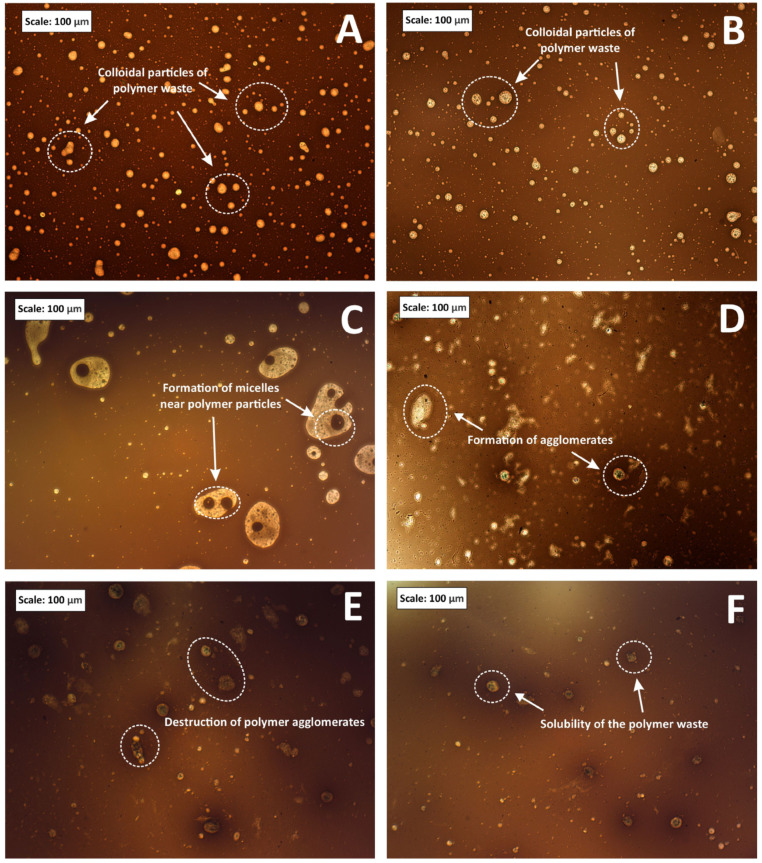
(**A**) LDPE 1.5% + SBS 1.5%, (**B**) LDPE 1.5% + Elvaloy 1%, (**C**) LDPE 2% + Elvaloy 1%, (**D**) HDPE 1.5%, (**E**) HDPE 1% + SBS 1.5%, and (**F**) HDPE 1.5% + SBS 1.5%.

Figure 3 shows that all bitumen samples exhibited smooth, even surfaces that appear to have an increased load-bearing capacity. With intensive mixing, bitumen components penetrate the polymer waste network and undergo polymerization under the influence of temperature. Bitumen modification with HDPE and LDPE polyethylene is a physical–chemical process involving polymer melting, dispersion, and swelling in the maltene phase of bitumen, forming a two-phase structure. Chemical polymerization or covalent bonding between the polyethylene and bitumen components did not occur, and the improved properties are due to the structure-forming and reinforcing effects of the polymer phase [30]. To increase the polymer waste content, condensation and cross-linking with alkyl, sulfide, or oxygen bridges are necessary, yielding large molecules that can form associates and organize into bundles due to the presence of π-conjugated systems and functional groups in their structure. The modifiers SBS and Elvaloy contain active sites for initiating the formation of new bonds during bitumen modification.

In all modified bitumen samples, dissolution of polymer waste in the hydrocarbon portion of the bitumen was observed. When bitumen is modified with polymer waste, all components within the bitumen undergo transformation, including oils, resins, and asphaltenes. Aromatic hydrocarbons in the oils participate in the dissolution of polymer particles. At the same time, resins participate in the structure formation of the polymer–bitumen binder by linking together aliphatic chains consisting of aromatic naphthenic and heterocyclic rings with alkyl side chains. Asphaltene molecules can be considered the condensation products of several resin molecules, which are less involved in the formation of new bonds. Polymer waste is characterized by a network structure that can be temporarily deformed and then returns to its original shape when exposed to external forces.

Samples A and B, with 1.5% LDPE, demonstrate complete dissolution of the polymer components. Sample A visually displays slightly more round dots, some of which are attributed to the SBS modifier. Increasing the LDPE concentration to 2% (sample C) leads to the formation of colloidal particles (micelles) that form a “core” solvated by polar bitumen molecules. Such a particle is called a complex structural unit of an oil-dispersed system and is found in a hydrocarbon environment. The obtained shell protects the polymer waste particles from further oxidation. Sample D demonstrates partial dissolution and the formation of agglomerates on the bitumen surface, where surface tension increases. Sample E presents the destruction of agglomerates; SBS leads to the formation of new bonds at the interface. Increasing the HDPE content to 1.5% in sample F led to increased solubility of the polymer waste, and new structures were observed. Detailed morphological analysis shows that the polymer waste dissolves in bitumen binders in the presence of modifiers.

**Figure 4:**
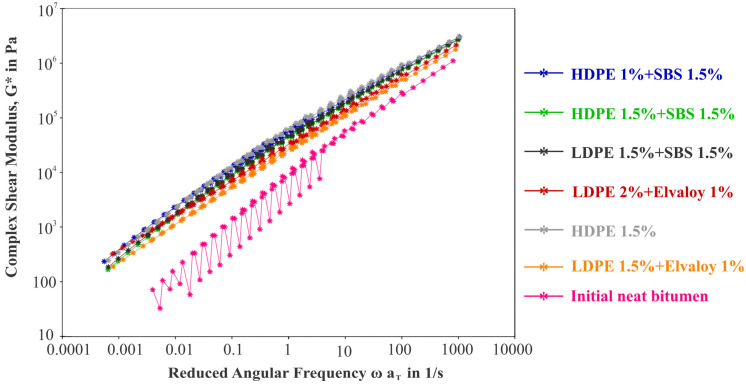
Master curve of complex shear modulus.

Figure 4 shows the master curve of the complex shear modulus versus reduced angular frequency at a reference temperature of 40 °C, constructed using the principle of temperature–frequency superposition (TFS). The graph shows that the modified bitumens exhibit significantly higher complex modulus values compared to the initial bitumen. This indicates an increase in stiffness and deformation resistance with the addition of polymer additives. The initial bitumen is located significantly below the other curves throughout the entire range of reduced frequencies, indicating its less structured structure and lower resistance to shear loads. The modified samples behave differently; they are located at the top of the graph and practically overlap. This indicates that modification with polyethylene significantly increases the complex modulus, forms a more rigid spatial structure, and improves bitumen resistance to high-temperature rutting resistance. The highest values are observed in systems with the addition of an SBS modifier, indicating a more pronounced reinforcing effect. The properties of the sample with Elvaloy are slightly lower than those of the SBS systems but significantly higher than those of the initial bitumen. This indicates that Elvaloy also increases rheological rigidity, but the structure is somewhat less rigid than in systems with polyethylene, which is consistent with the morphological analysis. On the other hand, the modified samples are characterized by better resistance to long-term changes and can increase the service life of asphalt concrete pavements, confirming the effectiveness of composite reinforcement.

**Figure 5:**
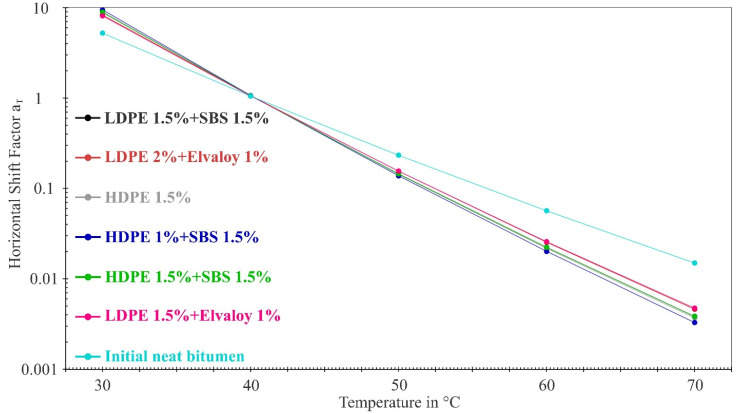
Curve of change in the horizontal shear coefficient depending on temperature.

A thermal sensitivity analysis based on the Williams–Landel–Ferry model (Figure 5) showed that the modified materials exhibit less temperature dependence than the typical behavior of viscoelastic materials. This is reflected in the flatter slopes of the curves. The modified sample with 1.5% HDPE and 1.5% SBS demonstrated the best thermal stability and the highest activation energy (172 kJ/mol), making it very suitable for regions with extreme weather conditions. The activation energy shown in Table 5 characterizes the energy barrier to viscoelastic processes, and the lower the value, the less energy required for them to occur. Modification with polymer waste indicates higher temperature sensitivity. The initial bitumen softens more easily with an increasing temperature and has a less stable structure. The addition of polymer waste results in the formation of a branched structure and stable thermal behavior in bitumen binders. Bitumen binders with HDPE exhibit high activation energies (168–172 kJ/mol), characterized by crystalline structures and more temperature-dependent behavior. The addition of SBS and HDPE enhances the temperature sensitivity and structure of HDPE but has little effect on LDPE and SBS. The Elvaloy modifier reduces the activation energy to 160 kJ/mol. The polymer–bitumen binder with Elvaloy effectively stabilizes the system and reduces temperature dependence. This means that samples with high activation energies are highly sensitive to temperature, while those with low activation energies are more stable across a wide temperature range.

**Figure 6:**
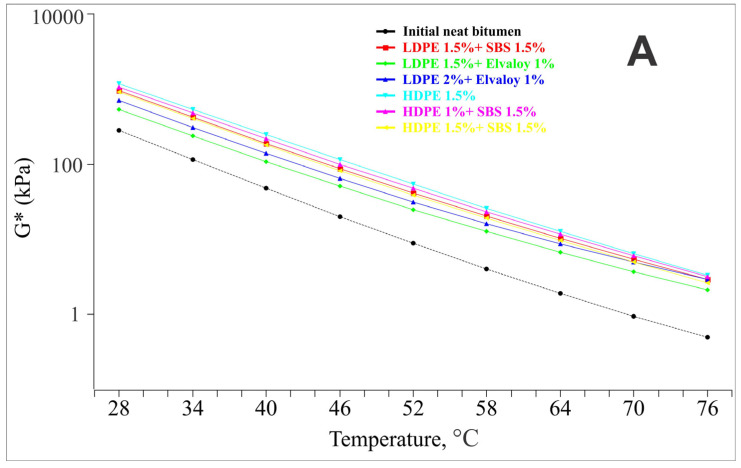

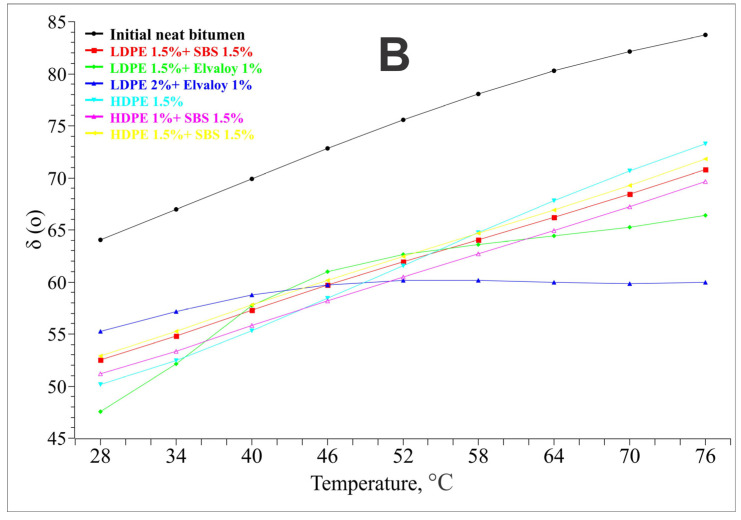
Complex modulus (**A**) and phase angle (**B**) of modified bitumen binders.

Figure 6 shows the G* and δ values of the bitumen binders as a function of temperature. With an increasing temperature and polymer waste content, the G* value of the modified binder decreases, while δ shows the opposite trend. It is worth noting that the G* and δ values for 1.5% LDPE and 1.5% SBS are very close to those for 1% HDPE and 1.5% SBS at different temperatures. This result suggests that the effect of LDPE on the degradation of bitumen binders will not significantly affect the deformation resistance of binders containing LDPE at temperatures of 4–76 °C, while the presence of the Elvaloy modifier leads to stabilization of the deformation resistance. This explanation can be related to the frequency rheological analysis.

The rheological analysis showed that these polymer wastes, when used in bitumen binders, exhibit relatively better deformation resistance and, consequently, higher rutting resistance at high temperatures. This is explained by the fact that a small amount of polymer waste leads to significant degradation and simultaneously promotes the formation of a new polymer network by cross-linking the partial degradation products, which facilitates increased temperature changes [31]. The results indicate that these formulations have the potential to increase the primary binder type to a higher PG value.

### Mechanical Characterization of Asphalt Concrete

To determine the effect of polymer waste on the properties of asphalt concrete, the composition of hot fine-grained dense asphalt concrete type B grade 1 was adopted. To determine the physical and mechanical properties of asphalt concrete based on bitumen binders modified with polymer waste, the following indicators, according to Kazakhstan standard 1225-2019 [32], were used (Table 6): average density, compressive strength at 20 and 50 °C, and crack resistance (tensile strength at splitting) at 0 °C. Asphalt concrete with 1.5% LDPE and 1.5% SBS shows a 8.1 and 44.5% increase in strength at 20 °C and crack resistance, respectively, compared to the addition of SBS. Asphalt concrete with 1.5% LDPE and 1% Elvaloy also presents high strength at 20 °C (1.67 MPa) and crack resistance (3.84 MPa) with a density of 2.23 g/cm^3^. A further increase in LDPE to 2% leads to improved physical and mechanical properties of asphalt concrete. In asphalt concrete, 1.5% HDPE shows low strength (1.92 MPa) and crack resistance (3.57 MPa). Use of the SBS and Elvaloy modifiers led to an increase in the physical and mechanical properties of asphalt concrete, increasing strength to 3.66 MPa and crack resistance by 19.6%, and in this case polymer waste acts as a reinforcing agent. The sample with 1.5% HDPE and 1.5% SBS shows a slight decrease in strength and crack resistance. All obtained samples containing polymer waste met the requirements of Kazakhstan standards.

Asphalt concrete as a temperature-sensitive pavement material is used on Kazakhstan national and local roads. The temperature stability of the bitumen and asphalt binder determines the durability of asphalt concrete and its resistance to low-temperature cracking and plastic deformation. In Kazakhstan only 70/100 and 100/130 grades of road bitumen are produced. In this case the main limitation of this study is that plastic material can be changed as their various mechanical and chemical properties can be influenced by the atmosphere, causing thermal and photo-oxidative degradation in Kazakhstan’s climate conditions (4 months of winter and 2 months of a hot summer season). In addition, our previous study [2] showed that using plasticizers in the modification of bitumen leads to compositions with poor adhesion to the mineral materials of the asphalt concrete mixture, because new phase appears that requires added stability compounds due to reduced chemical compatibility between the macromolecules and components of the asphalt concrete mixture. The results confirm that the modified bitumen complies with the requirements for polymer–bitumen binders of Kazakhstani standards and is suitable for the production of modified bitumen by its physical and chemical characteristics.

## 4. Conclusions

To reduce the cost of bitumen for road construction, the possibility of using polymer waste as a raw material for producing modified bitumen has been demonstrated. Several compositions were selected to meet the requirements of Kazakhstan standard 2534-2014 for polymer–bitumen binders based on polymer waste and modifiers like SBS and Elvaloy. A polymer–bitumen binder with 1.5% LDPE waste and 1.5% SBS meets the requirements of Kazakhstan standard 2534-2014 for BMP 50/70. A polymer–bitumen binder 2% LDPE waste and 0.5–1% Elvaloy meets the requirements of another Kazakhstan standard 2534-2014 for BMP 35/50. 1% HDPE and 1.5% SBS meet the requirements of Kazakhstan standard for BMP 35/50.

Rheological and morphological studies of the modified samples demonstrate high stiffness values, which potentially increases their resistance to deformation. This contributes to improved performance characteristics of the asphalt binder. However, it is recommended to test the performance characteristics of the corresponding mixtures on roads. A detailed study of this issue will open a new direction for recycling and the secondary use of polyethylene waste in durable asphalt concrete pavements.

Based on the obtained research results, asphalt concrete was prepared with an optimal polymer–bitumen binder formulation using plastic waste, which demonstrated a 3.4% increase in strength. This technology, using polymer waste in road construction, has a positive impact on global warming potential and energy efficiency, as it enables partial substitution of petroleum-based binders, reduces greenhouse gas emissions, and enhances the overall sustainability of pavement infrastructure. The use of polymer waste reduces the consumption of primary bitumen by 1 ton per 1 km of a standard road and bitumen modifier by 50%.

## Figures and Tables

**Table 1:** Physical–mechanical properties of virgin and secondary polyethylene (LDPE and HDPE).

Parameters	Virgin Polyethylene		Secondary Polyethylene	
	LDPE	HDPE	LDPE	HDPE
Tensile strength, MPa	16 ± 0.5	22–45 ± 0.5	8.8–10 ± 0.5	14–29 ± 0.5
Relative elongation at break, %	600–800	300–500	170–220	100–250
Brittleness temperature, °C	Under −70		From −40 to −50	From −30 to −40
Density, kg/m^3^	920 ± 0.01	941 ± 0.02	910 ± 0.1	938 ± 0.1
Melting point, °C	107 ± 2	127 ± 1	103 ± 2	120 ± 2

**Table 2:** Properties of polymer modifiers.

Indicators	SBS LG 501	Elvaloy
Density at 20 °C, g/cm^3^	0.94	0.94
Melt flow index at 200 °C/5 kg, g/10 min	0.5 g/10 min	8 g/10 min
Butadiene/styrene ratio, %	69/31	–
Melting point, °C	162	72.0
Maximum operating temperature, °C	260	280

**Table 3:** Physical and mechanical characteristics of polymer–bitumen binders with LDPE waste and SBS and Elvaloy modifiers.

Content, %			Softening Temperature, °C	Penetration Value at 25 °C	Ductility at 25 °C, cm
LDPE	SBS	Elvaloy			
1.5	–	–	63	39	27.5
2.0	–	–	66	43	17.5
1.0	1.0	–	60	44	29.5
1.5	1	–	68	41	16.5
2	1	–	58.2	51	33
1	1.5	–	61	48	29
1.5	1.5	–	62	51	30
2	1.5	–	61	45	18
1	2	–	61	45	25
1.5	2	–	61.4	43	28
2	2	–	62.5	46	25
1	–	0.5	71.8	37	15.5
1.5	–	0.5	65	35	22
2	–	0.5	66	31	17
1	–	1	64	36	23
1.5	–	1	65	38	26
2	–	2	65.3	36	22
Requirement of Kazakhstan standard 2534-2014 for BMP 50/70			not lower than 62	51–70	not less than 20
Requirement of Kazakhstan standard 2534-2014 for BMP 35/50			not lower than 65	35–50	not less than 15

**Table 4:** Physical and mechanical characteristics of polymer–bitumen binders with HDPE waste and SBS modifier.

Content, %		Softening Temperature, °C	Penetration Value at 25 °C	Ductility at 25 °C, cm
HDPE	SBS			
1		58	39	30
1.5	–	61.5	34	25
2.0	–	69.1	28	10
1.0	1.0	68.6	29	11.5
1.5	1	69	29	11
2	1	69.5	29	9.4
1	1.5	65.7	35	16
1.5	1.5	68	31	17
2	1.5	75	29	9.1
Requirement of Kazakhstan standard 2534-2014 for BMP 35/50		not lower than 65	35–50	not less than 15

**Table 5:** Activation energy of modified bitumen binders.

Sample	E_a_, kJ/mol
Initial bitumen	126.6
LDPE 1.5% + SBS 1.5%	167.5
HDPE 1% + SBS 1.5%	172.3
HDPE 1.5%	168.7
HDPE 1.5% + SBS 1.5%	167.6
LDPE 2% + Elvaloy 1%	161.9
LDPE 1.5% + Elvaloy 1%	160.9

**Table 6:** Physical and mechanical properties of asphalt concrete with polymer waste.

Composition of Asphalt Concrete	Density, g/cm^3^	Strength at 50 °C, MPa	Strength at 20 °C, MPa	Crack Resistance at 0 °C, MPa
Control sample	2.29	2.24	4.24	5.24
SBS 3%	2.23	1.26	3.21	3.46
Elvaloy 1%	2.21	1.37	3.08	3.93
LDPE 1.5% + SBS 1.5%	2.18	2.21	3.49	6.23
LDPE 1.5% + Elvaloy 1%	2.23	1.67	3.34	3.84
LDPE 2% + Elvaloy 1%	2.23	1.72	3.36	3.98
HDPE 1.5%	2.23	1.92	2.52	3.57
HDPE 1% + SBS 1.5%	2.25	2.32	3.66	4.44
HDPE 1.5% + SBS 1.5%	2.25	2.22	3.33	4.26
Kazakhstan standard 1225-2019 [32]	–	not less 1.3	not less 2.5	3.5–6.5

## Data Availability

The original contributions presented in this study are included in the article. Further inquiries can be directed to the corresponding authors.
